# Musculoskeletal Multibody Simulation Analysis on the Impact of Patellar Component Design and Positioning on Joint Dynamics after Unconstrained Total Knee Arthroplasty

**DOI:** 10.3390/ma13102365

**Published:** 2020-05-21

**Authors:** Maeruan Kebbach, Martin Darowski, Sven Krueger, Christoph Schilling, Thomas M. Grupp, Rainer Bader, Andreas Geier

**Affiliations:** 1 Biomechanics and Implant Technology Research Laboratory, Department of Orthopaedics, Rostock University Medical Center, 18057 Rostock, Germany; MartinMarkus.Darowski@med.uni-rostock.de (M.D.); rainer.bader@med.uni-rostock.de (R.B.); andreas.geier@med.uni-rostock.de (A.G.); 2 Aesculap AG Research and Development, 78532 Tuttlingen, Germany; sven.krueger@aesculap.de (S.K.); christoph.schilling@aesculap.de (C.S.); thomas.grupp@aesculap.de (T.M.G.); 3 Department of Orthopaedic Surgery, Ludwig Maximilians University Munich, Physical Medicine and Rehabilitation, Campus Grosshadern, 81377 Munich, Germany; 4 Department of Modern Mechanical Engineering, Waseda University, Tokyo 169-0072, Japan

**Keywords:** joint replacement, knee joint, total knee arthroplasty, patellar component, musculoskeletal multibody simulation, patellofemoral joint

## Abstract

Patellofemoral (PF) disorders are considered a major clinical complication after total knee replacement (TKR). Malpositioning and design of the patellar component impacts knee joint dynamics, implant fixation and wear propagation. However, only a limited number of studies have addressed the biomechanical impact of the patellar component on PF dynamics and their results have been discussed controversially. To address these issues, we implemented a musculoskeletal multibody simulation (MMBS) study for the systematical analysis of the patellar component’s thickness and positioning on PF contact forces and kinematics during dynamic squat motion with virtually implanted unconstrained cruciate-retaining (CR)-TKR. The patellar button thickness clearly increased the contact forces in the PF joint (up to 27%). Similarly, the PF contact forces were affected by superior–inferior positioning (up to 16%) and mediolateral positioning (up to 8%) of the patellar button. PF kinematics was mostly affected by the mediolateral positioning and the thickness of the patellar component. A medialization of 3 mm caused a lateral patellar shift by up to 2.7 mm and lateral patellar tilt by up to 1.6°. However, deviations in the rotational positioning of the patellar button had minor effects on PF dynamics. Aiming at an optimal intraoperative patellar component alignment, the orthopedic surgeon should pay close attention to the patellar component thickness in combination with its mediolateral and superior–inferior positioning on the retropatellar surface. Our generated MMBS model provides systematic and reproducible insight into the effects of patellar component positioning and design on PF dynamics and has the potential to serve as a preoperative analysis tool.

## 1. Introduction

Total knee replacement (TKR) is an established and effective surgical procedure for progressive osteoarthritis. TKR is currently performed over 700,000 times a year in the USA and this number is expected to grow exponentially worldwide [1,2]. Nevertheless, the rate of satisfied patients is 80%, which is rather low [2,3,4]. The patellofemoral (PF) joint represents a crucial part after total knee arthroplasty, and persistent PF pain remains a common postoperative complication with or without patellar resurfacing [5,6,7]. Complications include anterior knee pain, patellar maltracking, fracture, and patellar component loosening [8,9,10]. In this context, patella resurfacing is an important intraoperative factor: in the USA, more than 80% of primary TKRs are performed with this technique [11]. During surgery, the accurate positioning of the patellar component remains challenging because intraoperative alignment involves considerable inaccuracies [5,9,12,13]. The incidence of PF disorders ranges from 7% to 30% after a minimum of two postoperative years [14]. Patellar component malalignment is related to increased retropatellar loading and abnormal patellar kinematics [15,16]. Regarding the high number of intraoperative parameters, the dynamic interplay of the articulating joint partners of PF and tibiofemoral joint need to be analyzed in a systematic and reproducible manner [15,17,18]. 

Clinical observations alone cannot entirely explain unsatisfactory patient outcomes as they are often limited to the retrospective analysis of the influence of intraoperative parameters on pain and functional outcome [5,7,11,12,13,19,20]. However, clinical observations could be correlated with the mechanical loading of the joint to identify the underlying biomechanical causative chain. Therefore, knowledge of the biomechanical influence of intraoperative positioning of the patellar component during typical movements of daily living is essential to understand the underlying mechanical causes of implant failure and to improve the postoperative outcome. In this regard, the squat motion is known as one of the most dissatisfying motions after TKR [19].

Despite improvements in surgical instruments and techniques, many of the causes of revisions and patient dissatisfaction are directly related to implant component malalignment [6,18,21,22]. The morphology of the knee joint differs between both genders and different ethnicities [23,24,25,26]. These differences cannot only affect the implant component size selection but also the trochlear groove geometry, as well as the positioning of the patellar component between patients. Likewise, interindividual morphological joint variation is known to influence knee joint dynamics [27] and functionality of TKRs [26,28]. In this regard, Chen et al. [29] showed that the internal rotation of the femoral component and the varus malpositioning of the tibial component led to unfavorable postoperative loading conditions. Moreover, the malpositioning of the implant components often results in the overloading of the articulating joint compartments [18,30,31]. Different designs (dome-shaped, modified dome or anatomic design) and positions of the patellar button have been shown to affect knee joint dynamics [6,28,32,33,34,35,36,37]. An increased patellar component size was reported to affect the patellofemoral kinematics and postoperative outcome [38,39]. For instance, variations in the mediolateral and superior–inferior position of the patellar component resulted in different PF contact forces and kinematics [6,28,30,32,33,40,41]. Furthermore, the PF dynamics is influenced by the patellar component design [6,28] and the considerable impact of the patellar component thickness on PF kinematics and knee flexion has been widely reported [6,21,35,42,43,44,45]. For example, Abolghasemian et al. [43] reported a flexion loss of 1.28° with every millimeter of increased patellar component thickness. Bracey et al. [42] performed a similar study on 10 cadaveric knees and showed a flexion loss of 1.2° for each 2-mm increase of the patellar component thickness which resulted in a lateral patellar shift of more than 2 mm and a lateral patellar tilt of more than 4° whereas the patellar rotation remained nearly unchanged [43]. Bengs et al. [35] showed for 31 CR-TKRs with four different patellar thicknesses that on average the flexion was decreased by 3° for every 2-mm increase of the patellar component thickness but had no major impact on patella subluxation or tilt. Additionally, PF overstuffing has been reported with increased polyethylene wear, PF maltracking, and aseptic loosening of the patellar component [6,35,42,46,47]. Biomechanical studies have analyzed various mechanical aspects of patellar maltracking [15,32,33], e.g., medialization of the patellar component decreased the PF contact force [32,36,48,49] and altered the PF kinematics [36,41,48,50]. In a biomechanical study, Anglin et al. [48] found for a 2.5 mm component medialization a mean change in lateral patellar shift of 1.9 mm and a mean change in lateral tilt of 3.2°. These studies have contributed to understanding the positioning and design of the patellar component from a biomechanical perspective.

However, most experimental studies have only investigated passive knee flexion without active muscle forces, simulated rather uncommon motion patterns, commonly the knee rig configuration [28,33,42,51], or assumed a static quadriceps force, usually some predefined maximum value [28]. Furthermore, only a few studies addressing the biomechanical impact of different patellar component positions and designs on PF dynamics could be identified [15,18,28,33]. Instead, studies have revealed that the optimum position of the patellar component remains controversial [5,28,33,43,49], although evidently important for the postoperative outcome [28]. Some research groups have recommended medialization [8,41,48,50,52], while others have suggested centralization [32]. Moreover, biomechanical studies analyzing the superior–inferior position of the patellar component have reported contradictory findings [5,32,33,48], indicating a lack of understanding of this issue [5,49]. Hence, the PF joint mechanics have not yet been sufficiently quantified and understood so far [5,33]. The effects of various surgical parameters remain unclear and somewhat controversial. Using computational models, the influence of surgical and implant design parameters on knee joint dynamics can be investigated more comprehensibly [29,33,53,54,55,56].

Therefore, the current computational study aimed to systematically analyze and determine the biomechanical impact of patellar component malpositioning and design on PF dynamics during a dynamic squat motion using musculoskeletal multibody simulation (MMBS) in which a detailed knee joint model resembled the loading of a virtually implanted unconstrained cruciate-retaining (CR)-TKR including a dome patellar button. The findings could contribute to improving surgical techniques, preventing postoperative complications, and reducing wear propagation.

## 2. Materials and Methods

Our MMBS model is based on the experimental dataset of the *4th Grand Challenge Competition to Predict In Vivo Knee Loads* [57], which is a standardized dataset used by the research community to validate musculoskeletal models. This dataset comprises the CT scans (pre- and post-op) of a male subject (age = 88 years, height = 168 cm, and weight = 66.7 kg) who underwent TKR due to primary osteoarthritis and received an instrumented cruciate-retaining (CR) TKR (P.F.C. Sigma, DePuy Synthes, Raynham, MA, USA). This implant design was imported without changing the original standard size. The implanted TKR has a first-generation tray design eKnee and allows measuring the in vivo tibiofemoral contact forces during activities of daily living using an integrated telemetric force sensor. Concerning the knee implant, it represents a fixed-bearing, unconstrained bicondylar design which was implanted into the right knee of the patient. The femoral component had an asymmetrical dual radius design and was composed of cobalt–chromium alloy. The tibial component was composed of titanium alloy. Regarding the patellar component, an all-polyethylene dome-shaped component with three fixation pegs was used. The dataset enables the computational reconstruction of the 3D bone segments of the lower right extremity (pelvis, femur, patella, tibia, fibula, and pes), as well as the implant component geometries (femoral component, patellar button, tibial insert, and tibial tray). Moreover, motion capturing marker trajectories for activities of daily living are included, resulting in a comprehensive database for kinematic and kinetic model validation. A detailed description of this dataset can be found in Fregly et al. [57].

### 2.1. Overview of the Deployed Workflow of Musculoskeletal Multibody Simulation

**Figure 1:**
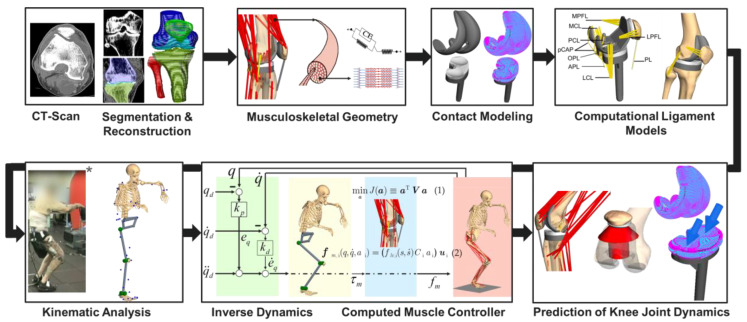
Workflow for generating the musculoskeletal multibody simulation model of the lower extremity with a total knee replacement. The illustration marked with * was taken from [57]. Permission to publish is granted under a CC BY open access license.

The workflow for developing our subject-specific MMBS model for the computational analysis of the PF joint after TKR is depicted in Figure 1. Briefly, the relevant bone geometries were reconstructed from preoperative CT scans [57,58], which allowed the virtual implantation of TKR implant geometries. The implementation of the musculoskeletal geometry was based on the data reported in the TLEM 2.0 anthropometric database [42]. Additionally, the origin and insertion of muscles and ligaments were verified by an experienced orthopedic surgeon. Contacting surfaces were modeled by means of the polygonal contact model [59] to enable physiological-like joint dynamics. In this regard, relevant ligament structures were modeled as sets of nonlinear force elements [60,61] to resemble the respective anatomy of the ligaments. The inverse kinematics analysis was performed on a realistic squatting motion as reported in the SimTK data set [57], in which recorded marker trajectories applied to a patient allowed the calculation of the relative joint coordinates in the MMBS model deploying a global optimization procedure [62,63]. Finally, an inverse dynamics analysis coupled with a static optimization [55,62,64,65,66] allowed the computation of the individual muscle forces for the forward dynamic prediction of knee joint dynamics, e.g., tibiofemoral and PF contact forces, tibiofemoral and PF kinematics, as well as muscle and ligament forces under the presence of surgical parameter variations related to TKR.

### 2.2. Musculoskeletal Multibody Simulation Model with a Cruciate-Retaining Total Knee Replacement

**Figure 2:**
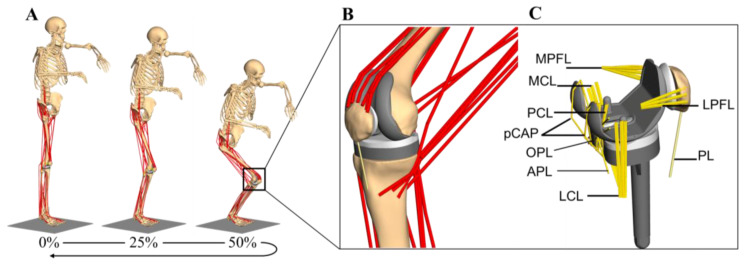
The developed musculoskeletal multibody simulation model with a cruciate-retaining total knee replacement in the lower right extremity during a dynamic squat motion combining musculoskeletal motion dynamics, knee implants with articular contact definitions, muscles, and ligaments (**A**). Detailed representation of the knee joint with implant components and muscle structures, including muscle wrapping. Note that ligaments are not shown for the sake of clarity (**B**). Investigated implant components with ligament structures of the tibio- and patellofemoral joint (**C**).

The MMBS model for the detailed analysis of the PF joint was generated based on a previously presented and validated MMBS model of the lower right extremity [65], Figure 2A,B. Implant and bone geometries, as well as relevant soft tissue structures, were modeled in the multibody software SIMPACK (V9.7, Dassault Systèmes Deutschland GmbH, Gilching, Germany). A variant of the computed muscle controller (CMC) with static optimization for individual muscle force prediction was implemented in MATLAB/Simulink^®^ (v8.1, 2013a, The MathWorks Inc., Natick, MA, USA) and interfaced the MMBS model via TCP/IP-communication for forward dynamic co-simulation. All simulations were performed on an off-the-shelf computer (Intel^®^ Xeon E5-1650 v4 CPU @3.60 GHz, 32 GB RAM).

The reconstructed bone segments were mutually connected by ideal joints as described in [62,65]. The respective centers of rotation of the ideal joints were determined by fitting cylinders or spheres into the cartilage surfaces of the articulating joint compartments. To ensure physiological-like roll-glide dynamics in the knee joint, both tibio- and patellofemoral joint compartments were modeled with six degrees of freedom (DoF) by implementing a polygon contact model [59], thereby resembling the complex articulation of the freeform implant surfaces. The lower left extremity was modeled as a symmetry condition in the sagittal plane. More precisely, the movement of the pelvis perpendicular to the sagittal plane was restrained by a spring-damper force element connecting the symmetry plane of the pelvis to the sagittal plane, thereby representing the lower left extremity during a symmetrical squat motion [65]. Regarding the squat motion, the patient started from a standing position and the range of knee flexion was about 0°–90°. The mass properties of the bone segments and soft tissue structures were calculated using regression equations as a function of the patient body weight [67].

For the description and comparison of the joint dynamics, standardized coordinate systems were established [68,69,70] which further allowed the identification of attachment points of relevant muscle and ligament structures as described in *Twente Lower Extremity Model 2.0* anthropometric database [71]. Additionally, the attachment sites of muscles and ligaments were verified by an experienced orthopedic surgeon. Muscles were implemented in the form of unidimensional Hill force elements [55,71,72]. The muscles were further subdivided into several structural bundles based on the attachment area to account for the wide attachment surfaces [71]. Muscle deflection phenomena around the bones were incorporated either as segment-fixed via points or using wrapping surfaces [73] where required. 

For a physiological representation of the knee joint dynamics, the MMBS model comprised, next to the explicit contact surface modeling of both the joint compartments, the implementation of all relevant ligamentous soft tissue structures (Figure 2C) with nonlinear force-strain relation (as nonlinear springs) [60,61]. Precisely, we implemented the posterior cruciate ligament (PCL), medial collateral ligament (MCL), lateral collateral ligament (LCL), oblique popliteal ligament (OPL), arcuate popliteal ligament (APL), posterior capsule (pCAP), medial patellofemoral ligament (MPFL), lateral patellofemoral ligament (LPFL), and the patellar ligament (PL) according to their anatomic descriptions, i.e., all ligaments were modeled as bundles of strands extending between the origin and insertion as described in Smith et al. [74]. The force elements for the representation of the ligaments followed a nonlinear elastic characteristic with a slack region as reported by Blankevoort et al. [61]. As it concerns the ligament parameterization, we first generated a MMBS model resembling the passive knee flexion, since the passive knee joint dynamics majorly depend on the ligaments and their parameterization (initial parameter sets were taken from the literature [61,74]). Then, by repeatedly simulating the knee flexion motion, the ligament parameters were adjusted with respect to the joint quantities for validation of the passive knee flexion. Once the appropriate ligament parameters were identified, the very same, parameterized ligament apparatus was transferred to the MMBS model resembling the squat motion and model validation was performed as described below (Section 2.5. *Validation of the musculoskeletal multibody simulation model*). Due to its high stiffness, the patellar ligament (PL) was modeled as a rigid coupling element between *Apex patellae* and *Tuberositas tibiae* with a fixed length [54,75]. The anterior cruciate ligament was virtually resected as it is sacrificed during CR-TKR surgery. A complete summary of the mechanical material properties for the ligaments [60,61,66,74] is provided in the Supplementary Information (Appendix A, Table A1). The PF joint was characterized by a patellar length of 43.6 mm, a patellar tendon length of 59.7 mm, and a tibial tuberosity-trochlear groove distance of 9.61 mm. The anthropomorphic details of the joint were in the normal range for Caucasians with native patellae [76,77]. Moreover, the retropatellar surface area after resection was in the normal range [78].

Hence, the developed MMBS model of the knee joint, allows for the systematic and reproducible analysis of the TF and PF joint dynamics with its emphasis on the detailed representation of the articulating endoprosthesis components and ligamentous structures during dynamic, muscle-induced full-body motion. 

### 2.3. Kinematic Analysis

The motion capture data comprises the trajectories of reflective skin markers applied to the patient’s body [57] as depicted in Figure 1. These marker trajectories were used as input for an optimization algorithm [79] to derive the generalized joint coordinates $q,\dot{q},\ddot{q}$ that describe the timely trajectories of the MMBS model’s DoF. More precisely, the skin markers were modeled as moving reference points and coupled to the respective anatomical landmark, i.e., related segment-fixed points on the bone surface using spring-damper force elements. In this manner, the inverse kinematics problem could be resolved by the minimization of the spring-damper potential, thereby minimizing the error between motion capture data and the MMBS model motion. The desired joint trajectories $q_{d},{\dot{q}}_{d},{\ddot{q}}_{d}$ were then used as input for the forward dynamic simulation of the muscle-induced squat motion for numerous MMBS model variations as described in the Section 2.6. *In silico study on the effect of the patellar component design and positioning on patellofemoral joint dynamics after TKR*. In this manner, we generated a nominal configuration of the MMBS model based on the optimal surgical technique [57] which was further used to predict different postoperative situations of the patellar component design and position. Finally, the implant configurations were verified by an experienced orthopedic surgeon.

### 2.4. Forward Dynamic Musculoskeletal Multibody Simulation of a Squat Motion

A variant of the CMC algorithm [66] with static optimization for individual muscle force prediction was implemented to track the desired joint coordinates, as derived from the experimental motion capture data, by generating coordinated muscle forces. This controller has been recently described and verified to enable in vivo knee kinematics and kinetics in patient-specific musculoskeletal models [55,65,66,74,80]. Within CMC, the inverse dynamics model serves for input-output linearization of the neuro-musculoskeletal system, which is further superposed with generic feedback control to accurately track the experimentally obtained motion maneuvers $q_{d},{\dot{q}}_{d},{\ddot{q}}_{d}$. Subsequently, the computed joint torques $\tau_{m}^{}$ necessary to drive the DoFs $q,\dot{q},\ddot{q}$ are distributed over the available muscle actuators $f_{m,i}^{}(\cdot )$ by means of static optimization [64]:

(1) $$\underset{a}{\min}J(a)\equiv a^{T}\;V\;a,\;\mathrm{subject}\;\mathrm{to}\;D\;a\;=\;\tau_{m}\;\mathrm{and}\;0\leq a_{i}\leq 1.$$

The aforementioned muscle distribution problem was solved for optimal muscle activation levels $a_{i}^{*}$ by minimizing an energy-optimal quadratic cost function J(a) in which the diagonal weight matrix $V=diag(V_{1},\ldots ,V_{n})$ includes the muscle volumes $V_{i}$ of each muscle unit and $a_{i}$ is bounded by its physiological limits $0\leq a_{i}\leq 1$. Moreover, the optimization problem is constrained by the set of linear equations $Da\;=\;\tau_{m}^{}$ where D represents the contribution of the respective muscle to the respective joint as described in a previous study [65]. We deployed a Hill-type muscle of the form:

(2) $$f_{m,i}(q,\dot{q},a_{i})=(f_{lv,i}(s,\dot{s})\;C_{i}\;a_{i})\;u_{i}\;\mathrm{with}\;C_{i}=A_{i}\;\sigma_{i}\;\mathrm{and}\;i=1,\ldots ,n,$$

where n is the number of muscle units, $f_{lv,i}(\cdot )$ is the force-length-velocity relation with muscle length s and muscle contraction velocity $\dot{s}$, $A_{i}$ describes the physiological cross-sectional area, $u_{i}$ is the muscle’s unit direction vector, and $\sigma_{i}$ is the maximal isometric muscle stress which was set to $\sigma_{i}=1\;\mathrm{Mpa}$ [72]. Note that within this work, the force-length-velocity factor was assumed to $f_{lv,i}(\cdot )=1$ as properties of the activated muscle structures and the activation dynamics have little influence on the prediction of muscle forces [64]. Therefore, the defined muscle force element depends on its theoretical maximum force $A_{i}\;\sigma_{i}$, its activation level $a_{i}$, and on the resulting moment arm only.

### 2.5. Validation of the Musculoskeletal Multibody Simulation Model

**Figure A1:**
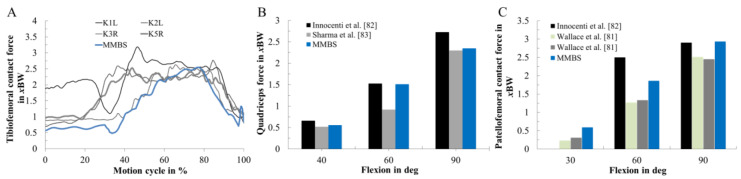
Validation of the musculoskeletal multibody simulation (MMBS) model. The MMBS model has been validated in terms of the tibiofemoral contact force (**A**), quadriceps force (**B**) and the patellofemoral contact force (**C**) during a dynamic squat motion. The tibiofemoral contact force is compared against the in vivo measurements of instrumented total knee replacements for three subjects (K1L, K2L, K3R, and K5R) [84]. The quadriceps force is compared against two simulation studies [82,83]. The resultant patellofemoral contact force is an important validation parameter for patellofemoral joint dynamics: it is compared against studies described in [81,82].

The predicted TF contact forces were validated using in vivo measured knee forces measured during squat motion exercises [57]. Furthermore, the TF contact force as well as the quadriceps force and PF contact force was compared with literature data [81,82,83,84] which is in Supplementary Information (Appendix B, Figure A1).

Since the in vivo measurements provided by the *4th Grand Challenge Competition to Predict In Vivo Knee Loads* [57] are limited to the contact forces acting in the TF joint, TF and PF kinematics were validated based on the in vitro and in silico results reported by Woiczinski et al. [85]. They measured TF and PF dynamics of 15 fresh frozen specimens with implanted CR-TKR during a squat motion using a knee rig [85]. 

### 2.6. In Silico Study on the Effect of the Patellar Component Design and Positioning on Patellofemoral Joint Dynamics after TKR

The validated MMBS model, as described previously, has been used as the nominal configuration corresponding to the optimal surgical technique [57] and served as a reference for subsequent model variations. Accordingly, with respect to the nominal model, we analyzed six patellar component configurations: spin of the patellar component ±5°, tilt of the patellar component ±5°, flexion-extension of patellar component ±5°, superior–inferior positioning of patellar component ±3 mm, and mediolateral positioning of patellar component ±3 mm. The position of the patellar component was changed based on a standardized coordinate system [70]. For instance, the shift was varied by medial or lateral movement of the center of the patellar component along a mediolateral axis defined as fixed to the patella. Moreover, we investigated the most important patellar button design parameter by increasing/decreasing the thickness of the patellar component ±2 mm. 

**Figure 3:**
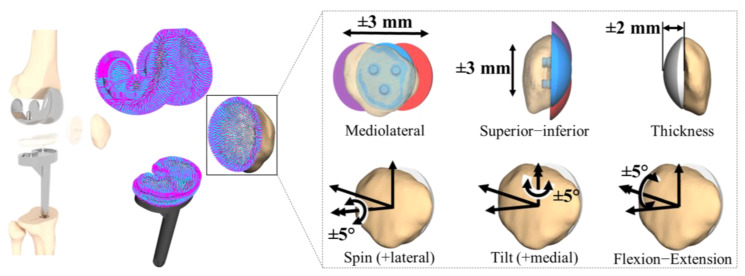
In silico study on the effect of the patellar component designs and positionings. Mediolateral positioning of patellar component ±3 mm, superior–inferior positioning of patellar component ±3 mm, and patellar button design parameter by increasing/decreasing the thickness of the patellar component ±2 mm. The rotational positioning was analyzed by the spin of the patellar component ±5°, tilt of the patellar component ±5°, and flexion-extension of patellar component ±5°.

The variations for each parameter configuration (Figure 3) were systematically chosen according to the reported findings from clinical [36,48,49], in vitro [15,16,32], and in silico [28,33] studies. The effect of each configuration was statistically evaluated with respect to the nominal simulation model as a function of the knee flexion angle over the full range of motion of the dynamic squat motion. Note that the calculated forces from the TF contact models were expressed with reference to the tibial component system to allow a direct comparison with the in vivo measured knee forces as reported by Fregly et al. [57].

### 2.7. Statistical Metrics

The predicted TF contact force of the MMBS model was compared with the in vivo measured knee force [57]. The prediction accuracy was quantified by mean absolute deviation (MAD) as a measure of magnitude differences, root-mean-square error (RMSE) as a measure for the difference between values predicted by the numerical model and the values actually observed from the experimental setup, Pearson correlation coefficient ($r^{2}$) as a measure of shape differences, and coefficient of determination ($R^{2}$) as a measure of magnitude and shape differences. For clarity, the mathematical definitions of the statistic metrics are provided in the Supplementary Information (Appendix C). 

## 3. Results

### 3.1. Validation of the Musculoskeletal Multibody Simulation Model

The validation of the TF contact force, as well as the quadriceps force and PF contact force using literature data [81,82,83,84], is presented in Supplementary Information (Appendix B, Figure A1).

**Figure 4:**
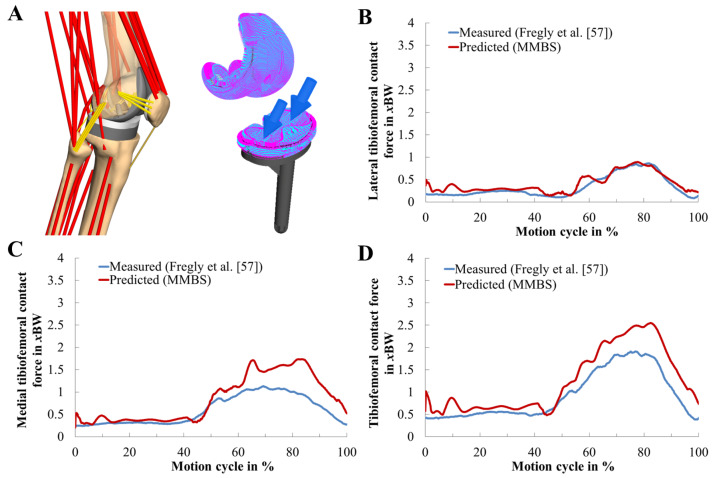
Musculoskeletal multibody simulation of the patellofemoral joint during the dynamic squat motion (**A**). Model validity was confirmed by comparing the reported lateral (**B**), medial (**C**), and total (**D**) tibiofemoral contact forces (in unit of body weight BW) of the in vivo measurements (blue, [57]) to our predictions (red).

The predicted and in vivo measured medial, lateral, and total TF contact forces during two-leg squat motion are depicted in Figure 4. Their quantification in terms of statistic metrics (Table 1) for the predicted contact forces of the MMBS model was in good agreement with a satisfactory level of accuracy (RMSE = 0.39 body weight (BW), $R^{2}$ = 0.94, $r^{2}$ = 0.97) in terms of the magnitude and the general trend. The medial contact force was slightly overestimated (RMSE = 0.35 BW). The predicted TF contact forces in the lateral compartment exhibited an excellent agreement (RMSE = 0.10 BW). Therefore, the MMBS model closely captured the overall pattern and timing ($R^{2}$ = 0.94, $r^{2}$ = 0.97) of the in vivo measured TF contact force, indicating sufficient model fidelity for contact force prediction.

**Figure 5:**
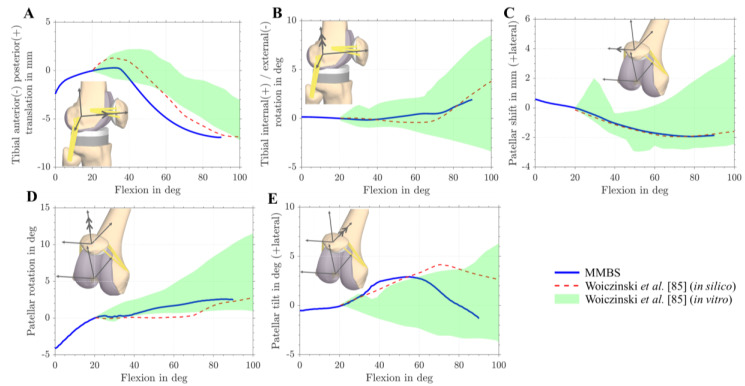
Tibio- and patellofemoral kinematics during dynamic squat motion. In silico (red dotted line) and in vitro (green area) [85] comparison of tibiofemoral and patellofemoral kinematics with kinematics obtained from musculoskeletal multibody simulation (MMBS) model (blue line). Comparison of anterior-posterior tibial translation with reference to the femur (**A**). Tibial internal/external rotation with reference to the femur (**B**). Patellar shift (**C**). Patellar rotation (**D**). Patellar tilt (**E**).

Moreover, the TF and PF kinematics were validated based on the in vitro and in silico results reported by Woiczinski et al. [85]. Specifically, anterior-posterior translation, tibial rotation, patellar tilt, patellar rotation, and patellar shift were compared (Figure 5). The PF and TF kinematics were reproduced with good agreement to the experimental and simulation data [85]. In general, the MMBS model predicted TF and PF kinematic patterns with a reasonable level of accuracy [85]. Overall, good agreement was observed between our MMBS model and reported data.

### 3.2. Effect of Patellar Component Design and Positioning on Patellofemoral Joint Dynamics after Unconstrained Total Knee Arthroplasty

**Figure 6:**
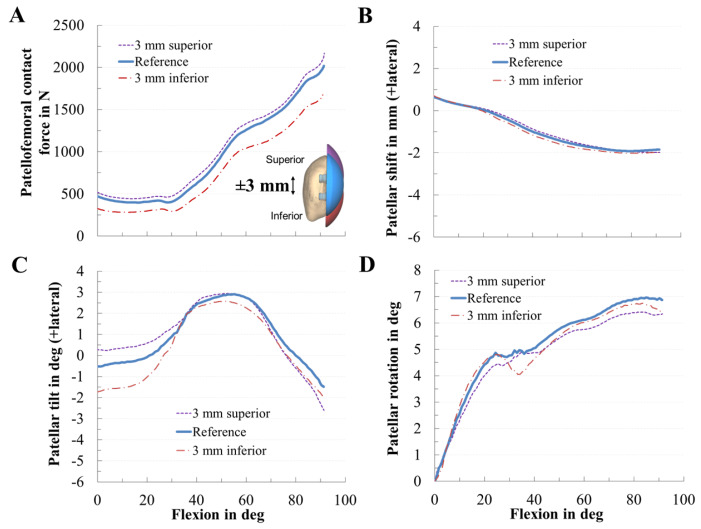
Effect of the superior–inferior positioning of the patellar component on patellofemoral dynamics. Effect of superior–inferior positioning of the patellar component on patellofemoral contact force (**A**), patellar shift (**B**), patellar tilt (**C**), and patellar rotation (**D**).

**Figure 7:**
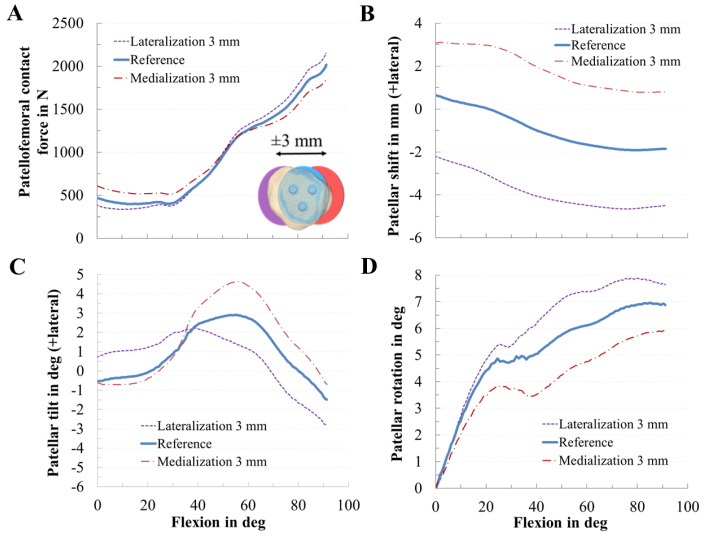
Effect of the mediolateral positioning of the patellar component on patellofemoral dynamics. Effect of mediolateral positioning of the patellar component on patellofemoral contact force (**A**), patellar shift (**B**), patellar tilt (**C**), and patellar rotation (**D**).

**Figure 8:**
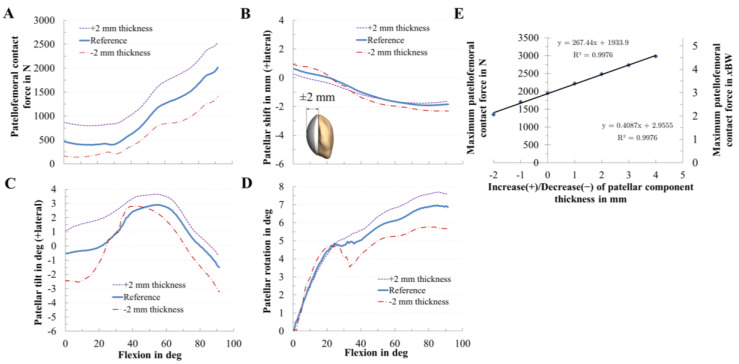
Effect of the patellar component thickness on patellofemoral dynamics. Effect of patellar component thickness on patellofemoral contact force (**A**), patellar shift (**B**), patellar tilt (**C**), and patellar rotation (**D**). Relationship of the maximum patellofemoral contact force for different patellar component thickness (**E**).

**Figure 9:**
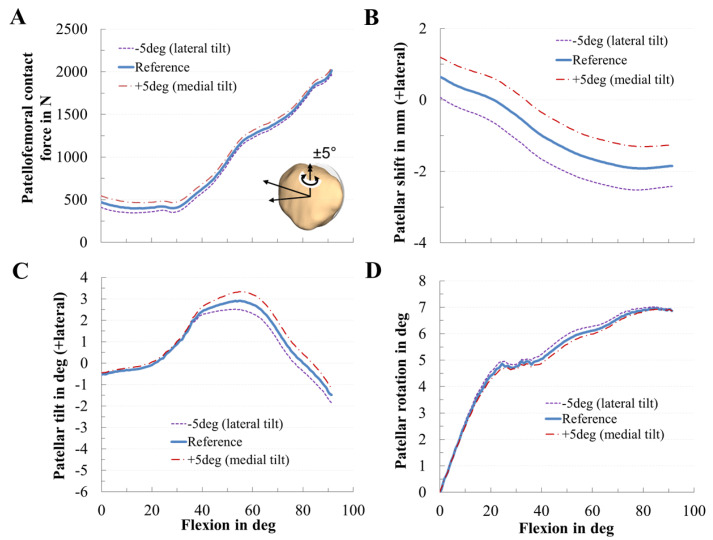
Effect of the tilting of the patellar component on patellofemoral dynamics. Effect of mediolateral positioning of the patellar component on patellofemoral contact force (**A**), patellar shift (**B**), patellar tilt (**C**), and patellar rotation (**D**).

**Figure 10:**
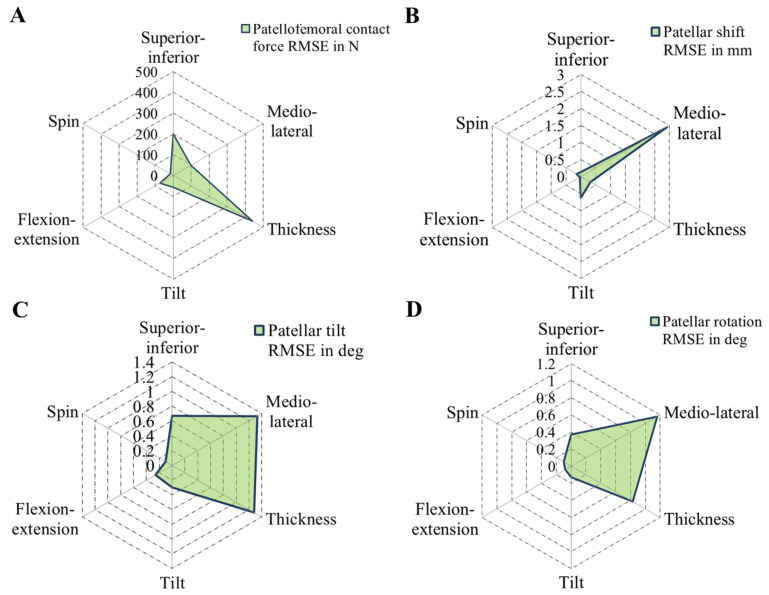
Radar chart representing the root-mean-square errors (RMSE) of patellofemoral contact force (**A**), patellar shift (**B**), patellar tilt (**C**), and patellar rotation (**D**) for different patellar component designs and positions: superior–inferior position, mediolateral position, patellar component thickness, tilt, flexion-extension, and spin.

PF dynamics was strongly affected by the position and design of the patellar component (Figure 6, Figure 7, Figure 8, Figure 9 and Figure 10). Generally, the PF contact forces increased with increasing knee flexion angle due to the progressive involvement of the *M. quadriceps femoris* that enables such deep flexion angles. However, depending on the positioning and design of the patellar component, it is possible to reduce or increase the PF contact force. In this context, the analysis showed that the PF contact force was strongly affected by the patellar component thickness (RMSE = 440 N), considerably affected by superior–inferior positioning (RMSE = 199 N), and only moderately affected by mediolateral (RMSE = 98 N) positioning (Figure 10). 

The variation in the superior–inferior positioning of the patellar component is depicted in Figure 6. An inferior position of the patellar component led to a reduction of the PF contact force (RMSE = 199 N) by up to 16% (Figure 6A). The maximum PF contact forces of the inferior, reference, and superior positions were 1626 N, 1947 N, and 2048 N, respectively. Superior positioning tended to cause a more medial tilting of the patella during flexion. An inferior position of the patellar component slightly increased the medial patellar shift, whereas a superior position of the patellar component decreased the lateral patellar tilt and shift (Figure 6B,C).

Similarly, the mediolateral positioning of the patellar component clearly affected the PF dynamics. Concerning the PF contact force, medialization and lateralization of the patellar component affected the PF contact forces (Figure 7A), i.e., a medialized patellar component decreased the maximum PF contact force during knee flexion by up to 8%. For instance, at 90° knee flexion, the PF contact force of the medial, reference, and lateral positions were 1798 N, 1947 N, and 2082 N, respectively. Furthermore, the mediolateral position of the patellar component clearly influenced PF kinematics regarding patellar shift, patellar tilt, and rotation. Mediolateral positioning of the patellar component shifted and tilted the patella path in the opposite direction, e.g., a medialized patella resulted in a more lateral shift and tilt of the patella and vice versa. Similarly, the patellar tilt was most sensitive to the mediolateral position of the patellar component. The more the patellar component was medialized, the more the patella tilted laterally with respect to the femur (Figure 7C).

The variation in the thickness of the patellar component dramatically changed the PF dynamics (Figure 8). Generally, lower PF contact force values were seen in thinner components, while with an increase in component thickness, the contact force increased by up to 27% (Figure 8A). For the nominal configuration, the peak PF contact force was 1947 N, and the corresponding value obtained from the other variations were 2480 N (+2 mm), and 1344 N (−2 mm). The contact force was 176% of that of the thinner component. There was a direct relationship between the maximum contact force and patellar component thickness (Figure 8E): an increasing component thickness leads to higher maximum contact forces following a linear trend (R² = 0.9976). Furthermore, we found that for every 1 mm of increased patellar thickness, PF contact force increased by 13.7% (0.41 BW). There was also an effect of the patellar component thickness on patellar tracking, e.g., an increased patellar component thickness led to an increased lateral patellar tilt (Figure 8C). Contrarily, the effect on the patellar shift was smaller (Figure 8B).

The rotational positioning of the patellar component influenced PF dynamics only slightly in our TKR design (Figure 9). Tilting the patellar component, however, corresponded to a nonsymmetrical rotation of the patellar dome and therefore affected patellar tilt and shift. A medial tilt of the patellar component slightly increased the PF contact force, the patella shifted, and tilted more laterally. Flexion-extension of the patellar component slightly affected the PF contact force, while the spin of the patellar component left the PF dynamics nearly unchanged (Figure 10).

### 3.3. Effect of Coupling Patellar Component Design and Positioning Parameters on Patellofemoral Joint Dynamics after Unconstrained Total Knee Arthroplasty

**Figure 11:**
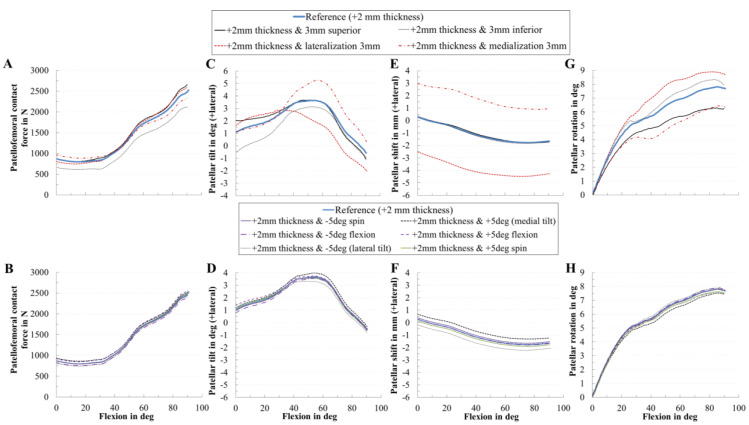
Effect of the increase of the patellar component thickness by +2 mm with all positioning parameters on patellofemoral dynamics. Effect of patellar component thickness combined with the positioning parameters on patellofemoral contact force (**A**,**B**), patellar tilt (**C**,**D**), patellar shift (**E**,**F**), and patellar rotation (**G**,**H**).

Additionally to the presented findings, we analyzed the coupling of the most critical patellar component design parameter with all other patellar component positioning parameters, i.e., we combined the increase of the thickness of 2 mm with all other positioning parameters (Figure 11). Similarly, the increase in the thickness combined with the rotational positioning of the patellar component influenced PF dynamics only slightly in our TKR design. However, an increase in the thickness combined with changing the mediolateral position of the patellar component mainly affected the PF kinematics. For instance, a solely increase in the thickness slightly changed the patellar shift (Figure 8B), whereas combined with a medialized patellar component the patellar shifted more laterally (Figure 11E). 

## 4. Discussion

In our computational study, we evaluated the effects of different intraoperative positionings and designs of the patellar component on both PF kinematics and kinetics during a dynamic squat motion of a subject with implanted CR-TKR with a dome patellar button. We developed a musculoskeletal knee joint model within a multibody simulation framework capable of analyzing the PF kinematics and kinetics. The results presented imply that even minor variations in the position and design of the patellar component can have a crucial effect on PF dynamics (Figure 6, Figure 7, Figure 8, Figure 9 and Figure 10). PF contact forces were mostly affected by patellar component thickness (up to 27%), patellar component superior–inferior positioning (up to 16%), and mediolateral patellar component positioning (up to 8%). Concerning PF kinematics, the patellar component thickness as well as the mediolateral position of the patellar component revealed the greatest impact. Based on our findings, we can conclude that the malalignment in mediolateral as well as superior–inferior direction and the thickness of the resurfaced patella are the most important intraoperative parameters affecting PF dynamics. Furthermore, we could show that the translational positioning is more critical than rotational positioning regarding the resulting PF contact force. Generally, our findings are in good agreement with those of previous experimental and clinical studies [5,6,28,32,35,36,48,49,86].

However, similar to other in vitro and in silico studies, our approach has some limitations. Our MMBS does not reflect a diverse population of different patients. Moreover, only the two-leg squat motion was considered. Additional simulations which cover more activities (e.g., rising from a chair, normal gait) will be helpful in the future for a more robust investigation and to support our presented findings as total knee kinematics are task-dependent [87]. However, the investigations were performed for a two-leg squat motion because this motion covers a wider range of knee flexion compared to gait and it is reported to be one of the most dissatisfying motions after total knee arthroplasty [19]. One limitation which should be considered when transferring our in silico findings to clinical practice is associated with ligament modeling as accurate representation of ligament parameters is still challenging, and ligaments’ mechanical properties might change during surgery. In the present study, the ligament parameters utilized were obtained from the literature. These assumptions might affect the prediction of joint dynamics. Likewise, the musculoskeletal geometry was based on the TLEM 2.0 anthropometric database [71]. It can be assumed that changes in the musculoskeletal geometry, e.g., muscle cross-sectional areas or muscle moment arms, of the muscles acting on the knee joint would have an effect on the forces and in turn kinematics of the PF joint. In addition, up to date, MMBS models hardly account for effects related to age or underlying conditions that might affect ligament or muscle structures. However, we believe that our MMBS model captures the physical condition of the examined subject well enough, as was made sure by rigorous model validation and diligent result analysis in cooperation with orthopaedic surgeons. The deployed methods are state-of-the-art and widely used in in silico models [29,33,53,75,80]. As explained above, our findings are based on one patient with one implant design and cannot be applied to all types of knee implants commercially available. Anatomical variability and further implant designs should be analyzed in future studies to support the reported findings. In future studies, different surgical alignment techniques (mechanical vs. kinematic), implant designs, different activities, and more subjects should be considered [37,38].

Orthopedic surgeons attempt to optimize the dynamic behavior of the knee joint by a correct intraoperative alignment of the patellar component. Interindividual morphological joint variation [23,24,25,26] is known to influence knee joint dynamics [27] and functionality of TKRs [26,28]. Furthermore, the difference in patellar component designs and the size of the patellar component are factors that influence the PF dynamics [28,37,38]. Regarding patellar resurfacing, the all polyethylene dome-shaped patellar component is currently the most common used design [8,28]. Therefore, our study covers the clinically most relevant TKR type. Our data indicate that the thickness of the analyzed dome-shaped patellar component affects PF contact forces, patellar tilt, and patellar rotation which has also been reported by other research groups [6,8,43]. In this regard, it was found that a higher retropatellar loading due to an increase in the thickness of the patellar component might contribute to anterior knee pain after TKR [6,44]. These higher forces can be explained by the increasing strain of the PF ligaments and can have a similar effect to overstuffing the PF joint which leads to anterior knee pain in some cases [8]. Although reduced patellar component thickness was beneficial due to a decreased PF contact force, it is important to avoid an excessively thin patellar component due to the risk of patellar maltracking and decreased PF ligament forces [46]. Our findings showed that, if the implant thickness increased, the lateral patellar tilt also increased which is in line with previous studies [21,42]. The influence of the thickness was not as high as reported by Bengs et al. [35]. Hsu et al. [44] reported that the PF contact force of the thicker patella was 174% higher compared to the thinner patella; in our study it was 176%. Some biomechanical studies [21,42,44,45] reported that overstuffing the PF joint induced higher lateral tilt of the patella which was also observed in our results. Additionally, a higher thickness might lead to increased polyethylene wear and aseptic loosening of the patellar component [35,42,46,47].

It has been reported that mediolateral positioning of the patellar component affects the PF dynamics [6,41]. We demonstrated the same in our study, i.e., a medialized patellar component reduced the resulting PF contact force during knee flexion in agreement with clinical observations [6,32,36,52,86]. Furthermore, medialization led to a more lateral path and tilt of the patella which is in line with previous findings [36,41,48]. A medialized patellar button decreases the Q-angle [8,41,86] and consequently lateralizes the patella bone, thereby leading to a higher lateral tilt [86]. Our results also indicate that the PF contact force decreased above 60° flexion due to medialization of the patella which might decrease postoperative pain [32,36,48]. In clinical studies, the incidence of lateral release was less for the medially placed patellar component [8,20,48,52]. However, we also showed that medialization caused a higher tendency for lateral tilting. The reported changes in shift, tilt, and PF forces are in good agreement with previous studies [20,48,52,86]. Concerning the patellar tilt, our data are comparable to a clinical study investigating the effects of medialization [50]. The reduction in the PF contact force due to medialization is consistent with a decrease in the Q-angle due to lateral shift of the patella [8,48].

To date, it is not clear whether the superior–inferior position of the patellar component influences knee dynamics after total knee arthroplasty [28,33,49]. We showed that the superior–inferior position of the patellar component affected the PF dynamics which is in line with previous studies [28,32,33]. In another study, Fitzpatrick et al. [28] identified the superior–inferior positioning as the most sensitive parameter using a dynamic finite element model supporting our findings. In this regard, our results, however, favor an inferior placement of the patellar component due to a decreased PF contact force. Nakamura et al. [33] recommended a superior placement of the patellar component, whereas other studies recommended an inferior placement [5,32]. As it concerns the PF contact forces, our results are contradictory to findings presented by Nakamura et al. [33] but consistent with other studies [5,30,32]. One explanation for the divergent data could be differences in the considered load cases and numerical simulation models used. The contradicting consequences of inferior position of the patellar component [33] occur only at high flexion angles (around 130° flexion) which were not reached in our analyzed load case. Furthermore, a higher number of PF ligaments were considered in our study. In general, inferior position of the patellar button might be favorable for daily living activities such as squatting but less desirable in patients who demand to reach higher flexion angles [88]. Based on our findings, the physiological reconstruction of the patellar position is of high importance. The most common conventional surgical techniques for patellar resection, i.e., freehand with a saw, using a saw guide, and a reamer reveal a high variance regarding the accuracy of the patellar cut. Although computer-navigation is used in TKR to improve the accuracy of the bony cut and component placement, currently it is focused on the positioning of the tibial and femoral component. However, studies with navigated patellar resurfacing systems showed an equal or higher accuracy for the patellar cut and the reconstructing of the physiological patellar position in comparison to conventional techniques with good short- and mid-term outcomes [12,13]. Our results emphasize the importance to focus on precise placement of the patellar component.

Another advantage of our study is the use of a detailed MMBS model incorporating relevant muscles of the lower extremity as active force elements compared to previous studies that assumed the muscles to apply only constant forces [15,16,30,51,85], considered only few muscles [28,33], or analyzed passive load cases without active muscle forces [34]. Our study showed the potential of MMBS in very comprehensively investigating the effects of surgical parameters on knee joint dynamics. The presented non-invasive method to simultaneously predict muscle, ligament, and knee joint forces can be used to improve the preclinical testing of TKRs as described by Affatato et al. [47] on the example of knee wear simulators. Computational data can provide additional insight into the influence of patellar component malpositioning on PF dynamics during active knee motions that commonly occur during daily living. Most orthopedic surgeons perform patellar bone preparation and component positioning without a navigation system [12,13]; however, small changes in the positioning leads to crucial changes in PF dynamics as demonstrated in this study. Furthermore, we showed that translational positioning of the patellar component is a relevant factor supporting the need for a navigation-based surgical procedure [12,13]. We pointed out that the patellar component is basically robust to rotational malalignment due to a consistent contact region between the articulating surfaces, which is in agreement with the findings of Fitzpatrick et al. [28]. Therefore, intraoperative placement of patellar components should first focus on translational position rather than rotational orientation.

## 5. Conclusions

In conclusion, the presented effects of patellar component design and positioning on PF kinematics and kinetics are in good agreement with previous experimental and computational studies. Aiming at an optimal intraoperative patellar component alignment, the most important parameters are component thickness, mediolateral and superior–inferior positioning. In both manual and navigation-based surgical techniques, the patellar thickness, patellar tracking, patellar position relative to the joint line, the orientation of the trochlear, and positioning of the anterior femoral component should be considered in order to prevent PF complications. Our findings will support orthopedic surgeons in intraoperative patellar component positioning from a biomechanical perspective. Regarding the different positioning of the patellar component, close attention should be paid to translational positioning as this might result in poor patient outcome.

## Figures and Tables

**Table 1:** Kinetic validation of the tibiofemoral joint. Quantitative analysis of the predicted medial, lateral, and total tibiofemoral contact forces with reference to in vivo measurements during the two-leg squat motion cycle as reported by Fregly et al. [57]. Units are expressed in Newton (N) and body weight (*x*BW). Mean absolute deviation (MAD), root-mean-square error (RMSE), Pearson correlation coefficient ($r^{2}$), and coefficient of determination ($R^{2}$).

Value	MAD [N]	MAD [*x*BW]	RMSE [N]	RMSE [*x*BW]	$r^{2}$	$R^{2}$
Total knee contact force	209.68	0.32	255.19	0.39	0.97	0.94
Medial contact force	163.02	0.25	226.65	0.35	0.94	0.89
Lateral contact force	46.67	0.07	68.28	0.10	0.95	0.91

## Appendix A

### Mechanical Material Properties of the Ligaments

**Table A1:** Mechanical properties (stiffness and reference strain) of the implemented ligaments were adapted from the literature [60,61,74,80]. The tibiofemoral ligament structures included: two bundles posterior cruciate ligament (PCL), two bundles lateral collateral ligament (LCL), three bundle medial collateral ligament (MCL), two bundles oblique popliteal ligament (OPL), one bundle arcuate popliteal ligament (APL), two bundles posterior capsule (pCAP), three bundles lateral patellofemoral ligament (LPFL), three bundles medial patellofemoral ligament (MPFL), and the patellar ligament (PL). Reference strains are the respective ligament strain values for the reference position upright standing. Stiffness is expressed in Newton per unit strain.

Tibiofemoral				Patellofemoral				
Ligament	Ligament Bundle	Stiffness (N)	Ref. Strain	Ligament	Ligament Bundle	Stiffness (N)		Ref. Strain
PCL	a	3000	−0.10	MPFL	p	2500		0.12
	p	1500	−0.03		c	2500		0.08
					d	2500		0.08
MCL	a	1500	0.04	LPFL	p	2000		0.06
					c	2000		0.06
					d	2000		0.06
	c	1500	0.04	PL		−		−
	p	1500	0.02	a			anterior	
LCL	a	2250	−0.25	c			central	
	p	2250	0.08	d			distal	
OPL	p	1250	0.06	i			inferior	
	d	1500	0.04	l			lateral	
pCAP	l	2500	0.05	m			medial/middle	
	m	2500	0.05	p			posterior/proximal	
APL		1500	0.04	s			superior	

## Appendix B

Investigated joint quantities during active two-leg squat motion for the validation of the musculoskeletal multibody simulation model (Figure A1). The tibiofemoral contact forces (Figure A1A) agreed with the measured contact forces in patients with telemetric TKR [84]. Likewise, the quadriceps force was in good agreement with previously published studies [82,83] (Figure A1B). Patellofemoral contact forces (Figure A1C) calculated at 30°, 60°, and 90° knee flexion were similar to findings by Wallace et al. [81] and Innocenti et al. [82].

## Appendix C

### Equations Used for Statistical Evaluation

The computational formulas for mean absolute deviation (*MAD*)

(A1) $$MAD=\frac{\sum_{i=1}^{n}\left|m_{i}-c_{i}\right|}{n},$$

root mean square error (*RMSE*),

(A2) $$RMSE=\sqrt{\frac{{\sum_{i=1}^{n}\left(m_{i}-c_{i}\right)}^{2}}{n}},$$

Pearson correlation coefficient ($r^{2}$)

(A3) $$r^{2}=\frac{n\sum m_{i}c_{i}-\sum m_{i}\sum c_{i}}{\sqrt{\left[n\sum m_{i}^{2}-{\left(\sum m_{i}\right)}^{2}\right]}\sqrt{\left[n\sum c_{i}^{2}-{\left(\sum c_{i}\right)}^{2}\right]}},$$

and coefficient of determination ($R^{2}$)

(A4) $$R^{2}={\left(r^{2}\right)}^{2},$$

presented here, where *m_i_* and *c_i_* represent the measured and calculated values at each time; step i, n is the total number of steps.

## References

[1] Kurtz S.M., Ong K.L., Lau E., Bozic K.J. (2014). Impact of the economic downturn on total joint replacement demand in the United States: Updated projections to 2021. J. Bone Jt. Surg. Am..

[2] Price A.J., Alvand A., Troelsen A., Katz J.N., Hooper G., Gray A., Carr A., Beard D. (2018). Knee replacement. Lancet.

[3] Bourne R.B., Chesworth B.M., Davis A.M., Mahomed N.N., Charron K.D.J. (2010). Patient satisfaction after total knee arthroplasty: Who is satisfied and who is not?. Clin. Orthop. Relat. Res..

[4] Pabinger C., Berghold A., Boehler N., Labek G. (2013). Revision rates after knee replacement. Cumulative results from worldwide clinical studies versus joint registers. Osteoarthr. Cartil..

[5] Assi C., Kheir N., Samaha C., Chamoun M., Yammine K. (2017). Novel anatomical-based surgical technique for positioning of the patellar component in total knee arthroplasty. SICOT J..

[6] Gasparini G., Familiari F., Ranuccio F. (2013). Patellar malalignment treatment in total knee arthroplasty. Joints.

[7] He J.-Y., Jiang L.-S., Dai L.-Y. (2011). Is patellar resurfacing superior than nonresurfacing in total knee arthroplasty? A meta-analysis of randomized trials. Knee.

[8] Matz J., Lanting B.A., Howard J.L. (2019). Understanding the patellofemoral joint in total knee arthroplasty. Can. J. Surg..

[9] Huang A.-B., Qi Y.-S., Song C.-H., Zhang J.-Y., Yang Y.-Q., Yu J.-K. (2016). Novel customized template designing for patellar resurfacing in total knee arthroplasty. J. Orthop. Res..

[10] Shah J.N., Howard J.S., Flanigan D.C., Brophy R.H., Carey J.L., Lattermann C. (2012). A systematic review of complications and failures associated with medial patellofemoral ligament reconstruction for recurrent patellar dislocation. Am. J. Sports Med..

[11] Fraser J.F., Spangehl M.J. (2017). International Rates of Patellar Resurfacing in Primary Total Knee Arthroplasty, 2004–2014. J. Arthroplast..

[12] Belvedere C., Ensini A., Tamarri S., d’Amato M., Barbadoro P., Feliciangeli A., Rao A., Leardini A. (2019). Does navigated patellar resurfacing in total knee arthroplasty result in proper bone cut, motion and clinical outcomes?. Clin. Biomech. (Bristol. Avon.).

[13] Fu C.K., Wai J., Lee E., Hutchison C., Myden C., Batuyong E., Anglin C. (2012). Computer-assisted patellar resection system: Development and insights. J. Orthop. Res..

[14] Narkbunnam R., Electricwala A.J., Huddleston J.I., Maloney W.J., Goodman S.B., Amanatullah D.F. (2019). Suboptimal patellofemoral alignment is associated with poor clinical outcome scores after primary total knee arthroplasty. Arch. Orthop. Trauma Surg..

[15] Steinbrück A., Schröder C., Woiczinski M., Schmidutz F., Müller P.E., Jansson V., Fottner A. (2017). Mediolateral femoral component position in TKA significantly alters patella shift and femoral roll-back. Knee Surg. Sports Traumatol. Arthrosc..

[16] Steinbrück A., Fottner A., Schröder C., Woiczinski M., Schmitt-Sody M., Müller T., Müller P.E., Jansson V. (2017). Influence of mediolateral tibial baseplate position in TKA on knee kinematics and retropatellar pressure. Knee Surg. Sports Traumatol. Arthrosc..

[17] Petersen W., Rembitzki I.V., Brüggemann G.-P., Ellermann A., Best R., Koppenburg A.G., Liebau C. (2014). Anterior knee pain after total knee arthroplasty: A narrative review. Int. Orthop..

[18] Steinbrück A., Schröder C., Woiczinski M., Müller T., Müller P.E., Jansson V., Fottner A. (2016). Influence of tibial rotation in total knee arthroplasty on knee kinematics and retropatellar pressure: An in vitro study. Knee Surg. Sports Traumatol. Arthrosc..

[19] Clement N.D., MacDonald D., Patton J.T., Burnett R. (2015). Post-operative Oxford knee score can be used to indicate whether patient expectations have been achieved after primary total knee arthroplasty. Knee Surg. Sports Traumatol. Arthrosc..

[20] Hofmann A.A., Tkach T.K., Evanich C.J., Camargo M.P., Zhang Y. (1997). Patellar component medialization in total knee arthroplasty. J. Arthroplast..

[21] Merican A.M., Ghosh K.M., Iranpour F., Deehan D.J., Amis A.A. (2011). The effect of femoral component rotation on the kinematics of the tibiofemoral and patellofemoral joints after total knee arthroplasty. Knee Surg. Sports Traumatol. Arthrosc..

[22] Schroer W.C., Berend K.R., Lombardi A.V., Barnes C.L., Bolognesi M.P., Berend M.E., Ritter M.A., Nunley R.M. (2013). Why are total knees failing today? Etiology of total knee revision in 2010 and 2011. J. Arthroplast..

[23] Wibeeg G. (1941). Roentgenographs and Anatomic Studies on the Femoropatellar Joint: With Special Reference to Chondromalacia Patellae. Acta Orthop. Scand..

[24] Mahfouz M., Abdel Fatah E.E., Bowers L.S., Scuderi G. (2012). Three-dimensional morphology of the knee reveals ethnic differences. Clin. Orthop. Relat. Res..

[25] Kim T.K., Phillips M., Bhandari M., Watson J., Malhotra R. (2017). What Differences in Morphologic Features of the Knee Exist Among Patients of Various Races? A Systematic Review. Clin. Orthop. Relat. Res..

[26] Ali A.A., Clary C.W., Smoger L.M., Dennis D.A., Fitzpatrick C.K., Rullkoetter P.J., Laz P.J. (2020). Computational framework for population-based evaluation of TKR-implanted patellofemoral joint mechanics. Biomech. Model. Mechanobiol..

[27] Fitzpatrick C.K., Baldwin M.A., Laz P.J., FitzPatrick D.P., Lerner A.L., Rullkoetter P.J. (2011). Development of a statistical shape model of the patellofemoral joint for investigating relationships between shape and function. J. Biomech..

[28] Fitzpatrick C.K., Baldwin M.A., Clary C.W., Wright A., Laz P.J., Rullkoetter P.J. (2012). Identifying alignment parameters affecting implanted patellofemoral mechanics. J. Orthop. Res..

[29] Chen Z., Wang L., Liu Y., He J., Lian Q., Li D., Jin Z. (2015). Effect of component mal-rotation on knee loading in total knee arthroplasty using multi-body dynamics modeling under a simulated walking gait. J. Orthop. Res..

[30] Luyckx T., Didden K., Vandenneucker H., Labey L., Innocenti B., Bellemans J. (2009). Is there a biomechanical explanation for anterior knee pain in patients with patella alta? Influence of patellar height on patellofemoral contact force, contact area and contact pressure. J. Bone Jt. Surg. Br..

[31] Kebbach M., Grawe R., Geier A., Winter E., Bergschmidt P., Kluess D., D’Lima D., Woernle C., Bader R. (2019). Effect of surgical parameters on the biomechanical behaviour of bicondylar total knee endoprostheses—A robot-assisted test method based on a musculoskeletal model. Sci. Rep..

[32] Lee T.Q., Budoff J.E., Glaser F.E. (1999). Patellar component positioning in total knee arthroplasty. Clin. Orthop. Relat. Res..

[33] Nakamura S., Tanaka Y., Kuriyama S., Nishitani K., Ito H., Furu M., Matsuda S. (2017). Superior–inferior position of patellar component affects patellofemoral kinematics and contact forces in computer simulation. Clin. Biomech. (Bristol. Avon.).

[34] Rosso I., Surace C., Antonaci P., Surace F.M., Negretto R.J. (2018). Influence of the patellar button thickness on the knee flexion after total knee arthroplasty. Acta Bioeng. Biomech..

[35] Bengs B.C., Scott R.D. (2006). The effect of patellar thickness on intraoperative knee flexion and patellar tracking in total knee arthroplasty. J. Arthroplast..

[36] Miller M.C., Zhang A.X., Petrella A.J., Berger R.A., Rubash H.E. (2001). The effect of component placement on knee kinetics after arthroplasty with an unconstrained prosthesis. J. Orthop. Res..

[37] Mochizuki T., Yano K., Ikari K., Hiroshima R., Okazaki K. (2020). Effect on patellar kinematics of the different patellar component designs in total knee arthroplasty: Intraoperative measurement of dome type versus anatomic type. Eur. J. Orthop. Surg. Traumatol..

[38] Joseph L., Batailler C., Roger J., Swan J., Servien E., Lustig S. (2020). Patellar component size effects patellar tilt in total knee arthroplasty with patellar resurfacing. Knee Surg. Sports Traumatol. Arthrosc..

[39] Atzori F., Sabatini L., Deledda D., Schirò M., Lo Baido R., Baido R.L., Massè A. (2015). Evaluation of anterior knee pain in a PS total knee arthroplasty: The role of patella-friendly femoral component and patellar size. Musculoskelet. Surg..

[40] van de Groes S.A.W., Koëter S., Waal Malefijt M., de Verdonschot N. (2014). Effect of medial–lateral malpositioning of the femoral component in total knee arthroplasty on anterior knee pain at greater than 8years of follow-up. Knee.

[41] Yoshii I., Whiteside L.A., Anouchi Y.S. (1992). The effect of patellar button placement and femoral component design on patellar tracking in total knee arthroplasty. Clin. Orthop. Relat. Res..

[42] Bracey D.N., Brown M.L., Beard H.R., Mannava S., Nazir O.F., Seyler T.M., Lang J.E. (2015). Effects of patellofemoral overstuffing on knee flexion and patellar kinematics following total knee arthroplasty: A cadaveric study. Int. Orthop..

[43] Abolghasemian M., Samiezadeh S., Sternheim A., Bougherara H., Barnes C.L., Backstein D.J. (2014). Effect of patellar thickness on knee flexion in total knee arthroplasty: A biomechanical and experimental study. J. Arthroplast..

[44] Hsu H.-C., Luo Z.-P., Rand J.A., An K.-N. (1996). Influence of patellar thickness on patellar tracking and patellofemoral contact characteristics after total knee arthroplasty. J. Arthroplast..

[45] Youm Y.-S., Cho W.-S., Woo J.-H., Kim B.-K. (2010). The effect of patellar thickness changes on patellar tilt in total knee arthroplasty. Knee Surg. Sports Traumatol. Arthrosc..

[46] Reuben J.D., McDonald C.L., Woodard P.L., Hennington L.J. (1991). Effect of patella thickness on patella strain following total knee arthroplasty. J. Arthroplast..

[47] Oishi C.S., Kaufman K.R., Irby S.E., Colwell C.W. (1996). Effects of patellar thickness on compression and shear forces in total knee arthroplasty. Clin. Orthop. Relat. Res..

[48] Anglin C., Brimacombe J.M., Wilson D.R., Masri B.A., Greidanus N.V., Tonetti J., Hodgson A.J. (2010). Biomechanical consequences of patellar component medialization in total knee arthroplasty. J. Arthroplast..

[49] Donell S. (2018). Patellar tracking in primary total knee arthroplasty. EFORT Open Rev..

[50] Kawano T., Miura H., Nagamine R., Urabe K., Matsuda S., Mawatari T., Moro-Oka T., Iwamoto Y. (2002). Factors affecting patellar tracking after total knee arthroplasty. J. Arthroplast..

[51] Didden K., Luyckx T., Bellemans J., Labey L., Innocenti B., Vandenneucker H. (2010). Anteroposterior positioning of the tibial component and its effect on the mechanics of patellofemoral contact. J. Bone Jt. Surg. Br..

[52] Lewonowski K., Dorr L.D., McPherson E.J., Huber G., Wan Z. (1997). Medialization of the patella in total knee arthroplasty. J. Arthroplast..

[53] Kuriyama S., Ishikawa M., Furu M., Ito H., Matsuda S. (2014). Malrotated tibial component increases medial collateral ligament tension in total knee arthroplasty. J. Orthop. Res..

[54] Tischer T., Geier A., Lenz R., Woernle C., Bader R. (2017). Impact of the patella height on the strain pattern of the medial patellofemoral ligament after reconstruction: A computer model-based study. Knee Surg. Sports Traumatol. Arthrosc..

[55] Geier A., Aschemann H., D Lima D., Woernle C., Bader R. (2018). Force Closure Mechanism Modeling for Musculoskeletal Multibody Simulation. IEEE Trans. Biomed. Eng..

[56] Affatato S., Ruggiero A. (2019). A Critical Analysis of TKR In Vitro Wear Tests Considering Predicted Knee Joint Loads. Materials.

[57] Fregly B.J., Besier T.F., Lloyd D.G., Delp S.L., Banks S.A., Pandy M.G., D’Lima D.D. (2012). Grand challenge competition to predict in vivo knee loads. J. Orthop. Res..

[58] Spitzer V.M., Whitlock D.G. (1998). The visible human dataset: The anatomical platform for human simulation. Anat. Rec..

[59] Hippmann G. (2004). An Algorithm for Compliant Contact Between Complexly Shaped Bodies. Multibody Syst. Dyn..

[60] Wismans J., Veldpaus F., Janssen J., Huson A., Struben P. (1980). A three-dimensional mathematical model of the knee-joint. J. Biomech..

[61] Blankevoort L., Kuiper J.H., Huiskes R., Grootenboer H.J. (1991). Articular contact in a three-dimensional model of the knee. J. Biomech..

[62] Herrmann S., Kluess D., Kaehler M., Grawe R., Rachholz R., Souffrant R., Zierath J., Bader R., Woernle C. (2015). A Novel Approach for Dynamic Testing of Total Hip Dislocation under Physiological Conditions. PLoS ONE.

[63] Leardini A., Chiari L., Della Croce U., Cappozzo A. (2005). Human movement analysis using stereophotogrammetry. Part 3. Soft tissue artifact assessment and compensation. Gait Posture.

[64] Anderson F.C., Pandy M.G. (2001). Static and dynamic optimization solutions for gait are practically equivalent. J. Biomech..

[65] Geier A., Kebbach M., Soodmand E., Woernle C., Kluess D., Bader R. (2019). Neuro-musculoskeletal flexible multibody simulation yields a framework for efficient bone failure risk assessment. Sci. Rep..

[66] Thelen D.G., Anderson F.C., Delp S.L. (2003). Generating dynamic simulations of movement using computed muscle control. J. Biomech..

[67] Winter D.A. (2009). Biomechanics and Motor Control of Human Movement.

[68] Wu G., Siegler S., Allard P., Kirtley C., Leardini A., Rosenbaum D., Whittle M., D’Lima D.D., Cristofolini L., Witte H. (2002). ISB recommendation on definitions of joint coordinate system of various joints for the reporting of human joint motion--part I: Ankle, hip, and spine. International Society of Biomechanics. J. Biomech..

[69] Grood E.S., Suntay W.J. (1983). A joint coordinate system for the clinical description of three-dimensional motions: Application to the knee. J. Biomech. Eng..

[70] Bull A.M.J., Katchburian M.V., Shih Y.-F., Amis A.A. (2002). Standardisation of the description of patellofemoral motion and comparison between different techniques. Knee Surg. Sports Traumatol. Arthrosc..

[71] Carbone V., Fluit R., Pellikaan P., van der Krogt M.M., Janssen D., Damsgaard M., Vigneron L., Feilkas T., Koopman H.F.J.M., Verdonschot N. (2015). TLEM 2.0—A comprehensive musculoskeletal geometry dataset for subject-specific modeling of lower extremity. J. Biomech..

[72] An K.N., Kaufman K.R., Chao E.Y. (1989). Physiological considerations of muscle force through the elbow joint. J. Biomech..

[73] Charlton I.W., Johnson G.R. (2001). Application of spherical and cylindrical wrapping algorithms in a musculoskeletal model of the upper limb. J. Biomech..

[74] Smith C.R., Vignos M.F., Lenhart R.L., Kaiser J., Thelen D.G. (2016). The Influence of Component Alignment and Ligament Properties on Tibiofemoral Contact Forces in Total Knee Replacement. J. Biomech. Eng..

[75] Marra M.A., Vanheule V., Fluit R., Koopman B.H.F.J.M., Rasmussen J., Verdonschot N., Andersen M.S. (2015). A subject-specific musculoskeletal modeling framework to predict in vivo mechanics of total knee arthroplasty. J. Biomech. Eng..

[76] Ortug A., Ormeci T., Yuzbasioglu N., Albay S., Seker M. (2018). Evaluation of normal tibial tubercle to trochlear groove distance in adult Turkish population. Niger. J. Clin. Pract..

[77] Schlenzka D., Schwesinger G. (1990). The height of the patella: An anatomical study. Eur. J. Radiol..

[78] Baldwin J.L., House C.K. (2005). Anatomic dimensions of the patella measured during total knee arthroplasty. J. Arthroplast..

[79] Herrmann S., Kähler M., Grawe R., Kluess D., Woernle C., Bader R., Flores P., Viadero F. (2015). Physiological-Like Testing of the Dislocation Stability of Artificial Hip Joints. New Trends in Mechanism and Machine Science: From Fundamentals to Industrial Applications.

[80] Thelen D.G., Won Choi K., Schmitz A.M. (2014). Co-simulation of neuromuscular dynamics and knee mechanics during human walking. J. Biomech. Eng..

[81] Wallace D.A., Salem G.J., Salinas R., Powers C.M. (2002). Patellofemoral joint kinetics while squatting with and without an external load. J. Orthop. Sports Phys. Ther..

[82] Innocenti B., Pianigiani S., Labey L., Victor J., Bellemans J. (2011). Contact forces in several TKA designs during squatting: A numerical sensitivity analysis. J. Biomech..

[83] Sharma A., Komistek R.D., Ranawat C.S., Dennis D.A., Mahfouz M.R. (2007). In vivo contact pressures in total knee arthroplasty. J. Arthroplast..

[84] Kutzner I., Heinlein B., Graichen F., Bender A., Rohlmann A., Halder A., Beier A., Bergmann G. (2010). Loading of the knee joint during activities of daily living measured in vivo in five subjects. J. Biomech..

[85] Woiczinski M., Steinbrück A., Weber P., Müller P.E., Jansson V., Schröder C. (2016). Development and validation of a weight-bearing finite element model for total knee replacement. Comput. Methods Biomech. Biomed. Eng..

[86] McPherson E.J. (2006). Patellar tracking in primary total knee arthroplasty. Instr. Course Lect..

[87] Schütz P., Postolka B., Gerber H., Ferguson S.J., Taylor W.R., List R. (2019). Knee implant kinematics are task-dependent. J. R. Soc. Interface.

[88] Mulholland S.J., Wyss U.P. (2001). Activities of daily living in non-Western cultures: Range of motion requirements for hip and knee joint implants. Int. J. Rehabil. Res..

